# “Worse With Sitting”: A Case of Severe Hypoxia Due to an Undiscovered Patent Foramen Ovale

**DOI:** 10.7759/cureus.79848

**Published:** 2025-02-28

**Authors:** Chan Woo Kim, Jonathan Weiss, Kaavya Mandi, Curtis A Hendrix, Gerardo Carino

**Affiliations:** 1 Internal Medicine, Warren Alpert Medical School, Brown University, Providence, USA; 2 Pulmonary and Critical Care Medicine, Warren Alpert Medical School, Brown University, Providence, USA

**Keywords:** atrial septal defect (asd), hypoxia, patent foramen ovale (pfo), platypnea orthodeoxia syndrome, transthoracic echocardiogram

## Abstract

Patent foramen ovale (PFO) is a cardiac shunt that results when the foramen ovale fails to close after birth. While it is relatively common, most cases are asymptomatic. The primary clinical implication of PFO is an increased risk of cryptogenic stroke. Accordingly, most literature addresses PFO through the lens of treating/preventing neurological disease. Much less research has been devoted to the minority of patients who suffer pulmonary and cardiovascular symptoms that are directly caused by their PFO. Here, we discuss a case of an elderly female patient presenting with hypoxia requiring high respiratory support, later found to be the result of a large, previously unknown PFO.

## Introduction

Patent foramen ovale (PFO) is a congenital atrial septal defect (ASD) that results when a remnant of fetal circulation, the foramen ovale, fails to close after birth [1,2]. In the fetus, a PFO allows oxygenated blood from the placenta to bypass the lungs and enter the fetal circulation. However, with birth, the PFO closes in order for the lungs to enter the circulation and be effective in blood oxygenation. Normally, an increase in the left atrial (LA) pressure and a decrease in pulmonary vascular resistance and right-sided cardiac pressures occur at birth. This results in the gradual closure of the flap of the foramen ovale against the atrial septum within the first two years of life [1,2]. PFO is the result of an incomplete closure. With incomplete closure, deoxygenated blood from the right side of the heart can be shunted to the left side of the heart, bypassing the pulmonary vasculature, which can result in systemic hypoxia. Usually, this remains asymptomatic throughout life because the pressure on the right atrial (RA) side is typically lower than pressures in the left atrium, so right to left blood flow does not occur. But in states when right to left flow does occur, it can result in an intracardiac shunt and hypoxia or even embolic phenomena of blood clots from the right side to the left, causing cryptogenic strokes.

The prevalence of PFO is approximately 25% in the general population, and most patients remain asymptomatic because the shunt remains closed under normal physiologic conditions [1,2]. However, certain hemodynamic conditions can cause the PFO flap to open wider than usual, allowing for the passage of thrombi or air into arterial circulation. This increases the risk of several conditions, including paradoxical brain embolism and systemic arterial embolism [3]. In certain cases, with an increase in the RA pressure, there can be a rapid increase in the right-to-left shunting of venous blood, leading to severe systemic hypoxia [1,3]. Here, we discuss a case of a 78-year-old patient with an unremarkable past medical history presenting with severe hypoxia with positional worsening that required high respiratory support, later found to be the result of a large, previously unknown PFO. This condition is known as platypnea-orthodeoxia syndrome (POS) and is an extremely uncommon presentation for a PFO.

## Case presentation

A previously healthy 78-year-old female patient presented to the emergency department for a sebaceous cyst on her back without any respiratory complaints. However, she was found to be hypoxic to 87% on 6 L nasal cannula, and this dropped dramatically with standing. Upon further questioning, the patient had had worsening dyspnea on exertion for two months and a history of a “heart murmur” since childhood. The patient continued to be hypoxic and was transitioned to high-flow nasal cannula (HFNC) with mild improvement. Her exam was notable for a continuous 2/6 systolic murmur near the cardiac base. When the patient’s hypoxia improved when lying flat, the physician care team began to suspect a PFO. There were no other notable physical exam findings.

The initial labs were remarkable for an arterial blood gas pH of 7.45 (7.38-7.42), pCO2 of 30 mmHg (35-45 mmHg), pAO2 of 80%, and a hemoglobin of 16 g/dL (12.1-15.1 g/dL) while on 10 L HFNC at 100% FiO2. The remainder of her labs were unremarkable. No acute cardiac or pulmonary abnormalities were seen in imaging. The initial EKG showed a right bundle branch block and left ventricular (LV) hypertrophy (unchanged from previous EKGs). Given the overall clinical picture of acute severe hypoxia and polycythemia also suggesting chronic hypoxia, the patient was admitted to the medical ICU (MICU) on 60 L HFNC for further management of a suspected PFO with POS. The patient was managed with permissive hypertension, given the potential protective effect against worsening shunt.

A transthoracic echocardiogram (TTE) showed a normal LV ejection fraction. It also revealed an asymmetric basal septal hypertrophy with concern for hypertrophic obstructive cardiomyopathy (HOCM) and LV outflow tract (LVOT) obstruction both at rest and while conducting the Valsalva maneuver (consistent with HOCM). A bubble study was conducted due to the suspicion of PFO and confirmed the suspected diagnosis of intracardiac shunt (Video 1).

**Video 1:** Echocardiogram of the patient undergoing a bubble study. Normal left atrium (LA) and left ventricle (LV) initially appear black (anechoic) and then become hypoechoic as the bubbles appearing in the right atrium (RA) and right ventricle (RV) move over via the patent foramen ovale (PFO) into the LV

While the TTE did visualize the PFO, there are frequent false negatives with TTE. In order to better visualize this, a follow-up TEE was conducted and showed an interatrial septal aneurysm and an anatomically large PFO with a large right-to-left shunting.

Subsequently, the patient underwent a right and left heart catheterization, which revealed a nonobstructive single-vessel coronary artery disease (CAD), a dynamic LVOT obstruction with a resting gradient of 50 mmHg, and also confirmed a PFO with a large atrial septal aneurysm. Pulmonary artery pressures were measured at 40/15 mmHg. The PFO was closed with a 35 mm Amplatzer PFO occluder (made by Abbott Cardiovascular in Plymouth, Minnesota, and the most widely utilized device to close PFOs) with intracardiac echo guidance. No residual shunt was evident by both color Doppler and agitated saline postdeployment. The patient was initiated on a dual-antiplatelet therapy for the observed CAD and a beta-blocker to reduce symptoms from the LVOT obstruction. Post procedure, the patient’s oxygen requirement rapidly decreased from 60 L to 2 L within the day. The patient was then transferred out of the MICU to the general medicine floor, where she remained stable on room air. She was discharged after two days with appropriate follow-up with the outpatient cardiology team.

## Discussion

Despite being extremely common (between 10% and 25% of the general population), the vast majority of PFOs are clinically asymptomatic. When present, symptoms may include stroke, migraines, and hypoxia. PFOs are most often found incidentally after a patient undergoes a screening TTE [3,4]. Depending on the size of the PFO, a heart murmur may be auscultated but can also be missed or deemed not clinically significant. There is some evidence supporting the closure of PFOs found among patients with cryptogenic strokes, but even among those patients, studies have shown that at least a third of PFOs are potentially incidental and not truly the cause of the stroke [5,6]. There have been efforts to identify “high-risk” PFOs based on size, “tunnel length” (measured through the max overlap of the septum primum and the septum secundum), and shunt severity (measured through the number of microbubbles seen on imaging). However, there remains limited data on the management of incidentally identified PFOs, and this complicates decisions regarding attempting to diagnose and treat PFOs [7]. The current consensus is to follow incidental PFOs until symptoms develop.

More unique to our case is that the patient presented with significant platypnea-orthodexia syndrome (POS), a phenomenon where a patient has normal oxygen saturation in a supine position but arterial hypoxemia when upright [4,8]. POS can be secondary to an intracardiac shunt (as with our patient), pulmonary vascular shunt, or ventilation-perfusion mismatch, which can change depending on the patient's position, when the patient is upright, or with any other changes in intrathoracic pressure. Other inciting factors, such as pulmonary embolism, acute pulmonary injury, or sequela after lung surgery, may result in an increase in the right-sided pressure, which then leads to increased right-to-left shunting and worse hypoxia [1,4,8]. Unfortunately, no such trigger was identified in our patient.

Our patient’s PFO was confirmed by TTE with bubble study, the current gold standard for the diagnosis of PFO [9,10]. While a TTE is sufficient for diagnosis, most patients undergo further workup, such as heart catheterization, to exclude other causes of dyspnea. Measuring the oxygen levels in the LV and the pulmonary vein by heart catheterization can confirm the presence of the shunt and rule out pulmonary hypertension, which can worsen after PFO closure [4]. It is unclear whether the incidentally found HOCM had any clinical significance in our case of POS due to PFO, and unfortunately, there is scant literature discussing any potential relationship.

The treatment for POS due to PFO is percutaneous or surgical elimination of the shunt. Our patient experienced a remarkable and rapid improvement in her hypoxemia after the procedure, which is consistent with observations in past studies. Most patients presenting with POS secondary to PFO had a near-complete resolution of dyspnea and hypoxemia following the procedure [11,12]. Of note, patients with pulmonary hypertension at baseline had less improvement of symptoms. Percutaneous PFO closure remains a safe procedure with minimal procedure-related complications. More recent studies show surgeries using newer devices, such as the Amplatzer PFO occluder used in our case, have complication rates of <1% [13,14]. Arrhythmias, such as supraventricular tachycardia or atrial fibrillations, were documented as possible complications but were largely transient and did not require any oral anticoagulation [13,14]. Overall, there was reduced recurrence of symptoms even in older patients like our case, providing evidence for the efficacy of the currently available PFO closure methods [15].

## Conclusions

PFO may remain clinically silent for years and is often discovered incidentally. Patients, such as the one discussed here, may present with a significant positional hypoxemia such as POS even without major respiratory complaints. This emphasizes the importance of considering measuring oxygenation in patients in both the seated and supine positions to screen for unrecognized POS. The presence of POS should raise suspicion for a PFO with acutely worsened right-to-left shunting. These patients should undergo a thorough dyspnea and hypoxemia workup, including lung imaging and echocardiography with bubble study. Considering the possibility of false negatives with TTE, TEE with bubble study and heart catheterization should be pursued when a high suspicion exists. 

The decision to undergo percutaneous closure of the PFO can be nuanced in many patients. While it is safe, it remains unclear whether asymptomatic patients would benefit. Current guidelines tend to focus on stroke prevention with PFO closure. Young patients with high-risk PFOs or large shunts may be considered for closure. In more elderly patients, the procedure has been found to be safe but unclear to have a stroke prevention benefit. However, in the cases of patients with significant hypoxia and symptoms due to a PFO, closure is the treatment of choice. Patients often experience rapid improvement in hypoxemia following the procedure with minimal complications, yet it remains important to follow up with the patient to monitor for complications or recurrence. Follow-up for our patient was recommended at one month, six months, one year, and then beyond, to include measurement of oxygen saturation and echocardiography to assess the function of the device and for any new shunts.
